# Glutamatergic Modulation of Brain Function in Psychosis: A Systematic Review of Neuroimaging Studies

**DOI:** 10.1016/j.bpsc.2025.07.004

**Published:** 2025-07-21

**Authors:** Ioana Varvari, Lara Bolte, Chiara Colli, Valentina Mancini, Matthew M. Nour, Philip McGuire, Robert A. McCutcheon

**Affiliations:** From the Department of Psychiatry, University of Oxford, Oxford, United Kingdom, Oxford Health NHS Foundation Trust, Oxford, United Kingdom; From the Department of Psychiatry, University of Oxford, Oxford, United Kingdom; From the Department of Psychiatry, University of Oxford, Oxford, United Kingdom, Department of Pathophysiology and Transplantation, University of Milan, Milan, Italy; Oxford Health NHS Foundation Trust, Oxford, United Kingdom, Wellcome Centre for Integrative Neuroimaging, Functional MRI of the Brain (FMRIB), Nuffield Department of Clinical Neurosciences, University of Oxford, Oxford, United Kingdom; From the Department of Psychiatry, University of Oxford, Oxford, United Kingdom, Oxford Health NHS Foundation Trust, Oxford, United Kingdom, Department of Psychosis Studies, King’s College London University, London, United Kingdom, Max Planck UCL Centre for Computational Psychiatry and Ageing Research, London, United Kingdom; From the Department of Psychiatry, University of Oxford, Oxford, United Kingdom, Oxford Health NHS Foundation Trust, Oxford, United Kingdom, Department of Psychosis Studies, King’s College London University, London, United Kingdom; From the Department of Psychiatry, University of Oxford, Oxford, United Kingdom, Oxford Health NHS Foundation Trust, Oxford, United Kingdom, Department of Psychosis Studies, King’s College London University, London, United Kingdom

## Abstract

Aberrant dopamine and glutamate signaling are implicated in the pathophysiology of schizophrenia. Existing treatments primarily target dopamine pathways underlying positive symptoms but have relatively little effect on cognitive and negative symptoms. Glutamatergic modulators may treat the latter symptom domains, and neuroimaging studies have the potential to identify therapeutic mechanisms. We conducted a systematic review to examine functional neuroimaging studies of glutamatergic modulators in psychosis and determine whether these agents alter brain activity, chemistry, or functional connectivity and whether such changes map onto clinical outcomes. Following Preferred Reporting Items for Systematic Reviews and Meta-Analyses (PRISMA) guidelines (PROSPERO: CRD42024549120), MEDLINE, Embase, and PsycInfo were searched from inception to June 2024 for studies administering pharmacologic glutamate modulators to individuals with psychosis, using functional neuroimaging (proton magnetic resonance spectroscopy [^1^H-MRS], functional magnetic resonance imaging [fMRI], arterial spin labeling, positron emission tomography, electroencephalography [EEG], or magnetoencephalography). Twenty-seven articles met inclusion criteria, encompassing 841 participants. Evidence from ^1^H-MRS suggests that sarcosine, *N*-acetylcysteine, and riluzole reduce glutamate concentrations in frontal and hippocampal regions, but clinical outcomes have not been investigated. Resting-state and task-based fMRI studies suggest that NMDA receptor modulators may normalize measures of functional dysconnectivity, although effects were often short-lived and did not always correspond to sustained symptom improvements. Similarly, EEG studies consistently identified normalization of mismatch negativity and gamma oscillations, but correlations with symptom or cognitive outcomes were inconsistent. While glutamatergic modulators show measurable effects on brain chemistry and electrophysiology, the relationship to robust, durable clinical benefits remains elusive. Future work should use larger, longer-duration, and multimodal imaging studies to clarify the precise mechanisms, optimal dosing, and the patient subgroups most likely to benefit from glutamatergic interventions in psychosis.

Schizophrenia is a chronic and severe disorder associated with 3 core symptom domains: positive (e.g., delusions, hallucinations, thought disorganization), negative (e.g., blunted affect, avolition, social withdrawal), and cognitive (e.g., deficits in executive functioning, attention, and learning) (1–3). It imposes a significant burden on individuals and health care systems worldwide; however, despite its impact, treatment outcomes remain limited (4,5). Most currently licensed treatments target the dopamine D_2_ receptor. While these are often effective treatments for positive symptoms, they fail to address negative and cognitive symptoms or sufficiently benefit the one-third of patients with treatment-resistant or ultratreatment-resistant forms of the illness (6–8). Addressing these issues is essential, as they are key drivers of functional outcomes and major contributors to societal and economic burdens (9,10).

Given these shortcomings, there is a pressing need to explore nondopaminergic pathways that could address cognitive, negative, and treatment-resistant positive symptoms. One potential approach is glutamatergic modulation. Glutamate (Glu) is the primary excitatory neurotransmitter in the central nervous system and exerts its effects through both ionotropic and metabotropic receptors (Figure 1A). Ionotropic Glu receptors, including NMDA receptors (NMDARs), AMPA receptors (AMPARs), and kainate receptors, mediate fast excitatory synaptic transmission via ligand-gated ion channels. In contrast, metabotropic Glu receptors (mGluRs) regulate neuronal excitability and synaptic plasticity through slower G protein–coupled signaling pathways (11).

Multiple lines of evidence from animal studies, genetic models, postmortem analyses, and neuroimaging suggest that glutamatergic dysfunction may contribute to cognitive and negative symptoms and treatment-resistant positive symptoms (3,12). The most consistent evidence concerns the NMDAR (13,14). Cognitive dysfunction is associated with NMDAR hypofunction in key cortical regions, including the prefrontal cortex (PFC) (15,16), hippocampus, and anterior cingulate cortex (ACC) (17,18) and subcortical regions including the basal ganglia and thalamus (19,20). Additionally, glutamatergic dysregulation may influence dopaminergic pathways, thereby exacerbating positive symptoms (21–24). Beyond NMDAR hypofunction, AMPAR (25,26), mGluR (27,28), and Glu and glycine transport systems (29–31) have also been implicated in schizophrenia pathophysiology.

Thus, targeting the Glu system offers a promising strategy for developing more symptomatically comprehensive schizophrenia treatments. However, there are no approved gluta-matergic treatments, with several phase 3 trials having failed to show significant benefit (3,32). Direct modulators investigated include mGlu_5_ positive allosteric modulators (e.g., ADX47273), glycine, D-serine, and memantine, which have shown efficacy in preclinical and small-scale trials but have rarely been tested in larger trials (28,33–35). Indirect modulators, including glycine transporter type 1 (GlyT1) inhibitors such as bitopertin and sarcosine as well as mGlu_2/3_ agonists such as LY2140023, initially demonstrated promise but ultimately faced challenges with clinical efficacy and safety (36–38). Similarly, iclepertin, also a GlyT1 inhibitor, failed to demonstrate any clinical benefit in large-scale studies (39).

To refine drug development efforts, we need greater mechanistic understanding into the specific effects of glutamatergic drugs. Here, functional and molecular neuroimaging, such as functional magnetic resonance imaging (fMRI), electroencephalography (EEG), and proton magnetic resonance spectroscopy (^1^H-MRS), represents one promising avenue to index glutamatergic modulation noninvasively in the living human brain. In addition to yielding mechanistic insight, this approach may also identify biomarkers indicating an increased likelihood of therapeutic response.

The aims of the current review were 1) to identify and describe how glutamatergic modulation influences brain function in patients with a psychotic disorder, 2) to identify and describe how glutamatergic modulation of brain function differs between patients and healthy control (HC) participants, 3) identify whether any factors (illness phase, sex, ethnicity) moderate the effects of glutamatergic modulation on brain function, and 4) examine associations between these neuroimaging measures and clinical outcomes.

## Methods

We followed Preferred Reporting Items for Systematic Reviews and Meta-Analyses (PRISMA) guidelines and registered the study on PROSPERO in June 2024 (CRD42024549120). MEDLINE, Embase, and PsycInfo databases were searched from inception to June 2024 for studies using EEG, magnetoencephalography (MEG), MRI, arterial spin labeling (ASL), MRS, or positron emission tomography (PET) to investigate the effects of Glu modulators in individuals with psychosis. See the Supplement for the full search string. To qualify for inclusion, studies must have been published in English within peer-reviewed journals and reflect the following population (psychosis, first-episode psychosis, schizophrenia, schizophreniform disorder, schizoaffective disorder), intervention (Glu-modulating pharmacological challenge), comparator (placebo or HC participants), and outcome (measures of neuroimaging brain function such as MRS measures of glutamate concentrations, fMRI measures of activity or connectivity, ASL measures of cerebral blood flow, EEG/MEG measures of event-related potentials of power spectra, etc.). Studies that were not original research, did not involve human participants, or focused exclusively on HC participants or clinical outcomes were excluded. A narrative synthesis as opposed to a meta-analysis was used given the heterogeneity of interventions and study designs (40). The Joanna Briggs Institute critical appraisal tools (41,42) were used for risk of bias appraisal (see the Supplement).

## Results

Twenty-seven articles with a combined sample size of 841 participants met the inclusion criteria following screening. The title and abstract screenings were performed independently by 4 reviewers (IV, LB, CC, and VM), with assignments being shuffled among them to facilitate reconciliation. Full-text screening for eligible studies was done by 2 reviewers (IV and CC) and reconciliated by VM. RAM reviewed each screening stage to ensure consistency. See Figure 2 for the PRISMA flowchart. The findings were structured first by neuroimaging modality to provide a technique-specific understanding of glutamatergic modulation and its associations with clinical, functional, and cognitive outcomes (Table 1).

**Figure 1:**
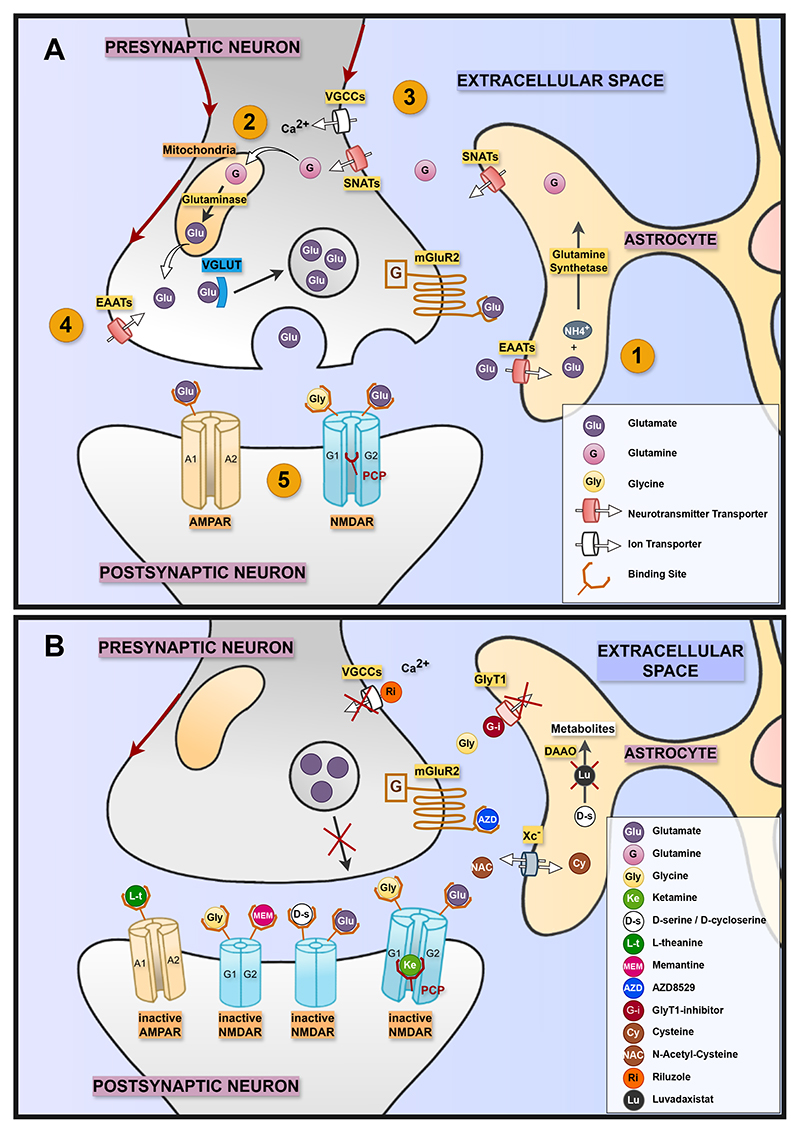
Glutamate at the synapse. **(A)** The glutamatergic synapse. 1) Glutamine biosynthesis: astrocytic EAATs take up glutamate from the synaptic cleft. Glutamate combines with NH4^+^ and is converted into glutamine-by-glutamine synthetase. Glutamine is released into the extracellular space via SNATs for neuronal uptake. 2) Glutamate–glutamine cycle: neuronal SNATs transport glutamine into the presynaptic terminal, where PAG converts it back to glutamate. Glutamate is packaged into synaptic vesicles by VGLUT for release. 3) Neuronal activation and glutamate release: Action potentials depolarize the presynaptic terminal (red arrows), opening VGCCs, and Ca^2+^ influx triggers vesicle release. 4) Glutamate reuptake and receptor binding: Released glutamate is cleared by EAATs back into astrocytes and neurons to sustain the cycle. Remaining glutamate binds to presynaptic and postsynaptic receptors: AMPARs, NMDARs, and mGluRs. 5) Postsynaptic receptor activation: AMPARs (GluA1-4) depolarize the postsynaptic membrane, facilitating NMDAR (GluN1/2) activation. NMDAR activation also requires binding by glutamate and glycine co-agonist. **(B)** Depiction of identified modulators mechanism of action. 1) Direct modulators. L-theanine and ketamine antagonize glutamate binding sites at AMPAR and NMDAR, respectively. D-serine and D-cycloserine are agonists at the glycine binding site of the NMDAR. Ketamine binds the PCP binding site within the NMDAR and prevents activation. AZD8529 binds mGluR and present vesicle fusion and Glu release. 2) Indirect modulators. Sarcosine, iclepertin, and PF-03463275 inhibit the GlyT1 transporter, increasing synaptic glycine levels. NAC enhances the Xc transport system by increasing the amount of cysteine, while riluzole blocks the VGSCs. Luva-daxistat inhibits DAAO, preventing the breakdown of D-serine and increasing its availability at the synaptic cleft. AMPAR, AMPA receptor; DAAO, D-amino acid oxidase; EAAT, excitatory amino acid transporter; LTP, long-term potentiation; mGluR, metabotropic glutamate receptor; NMDAR, NMDA receptor; PAG, phosphate-activated glutaminase; PCP, phencyclidine; SNAT, sodium-coupled neutral amino acid transporter; VGCC, voltage-gated calcium channel; VGLUT, vesicular glutamate transporter; VGSC, voltage-gated sodium channel; Xc system, cystine/glutamate antiporter system.

**Figure 2:**
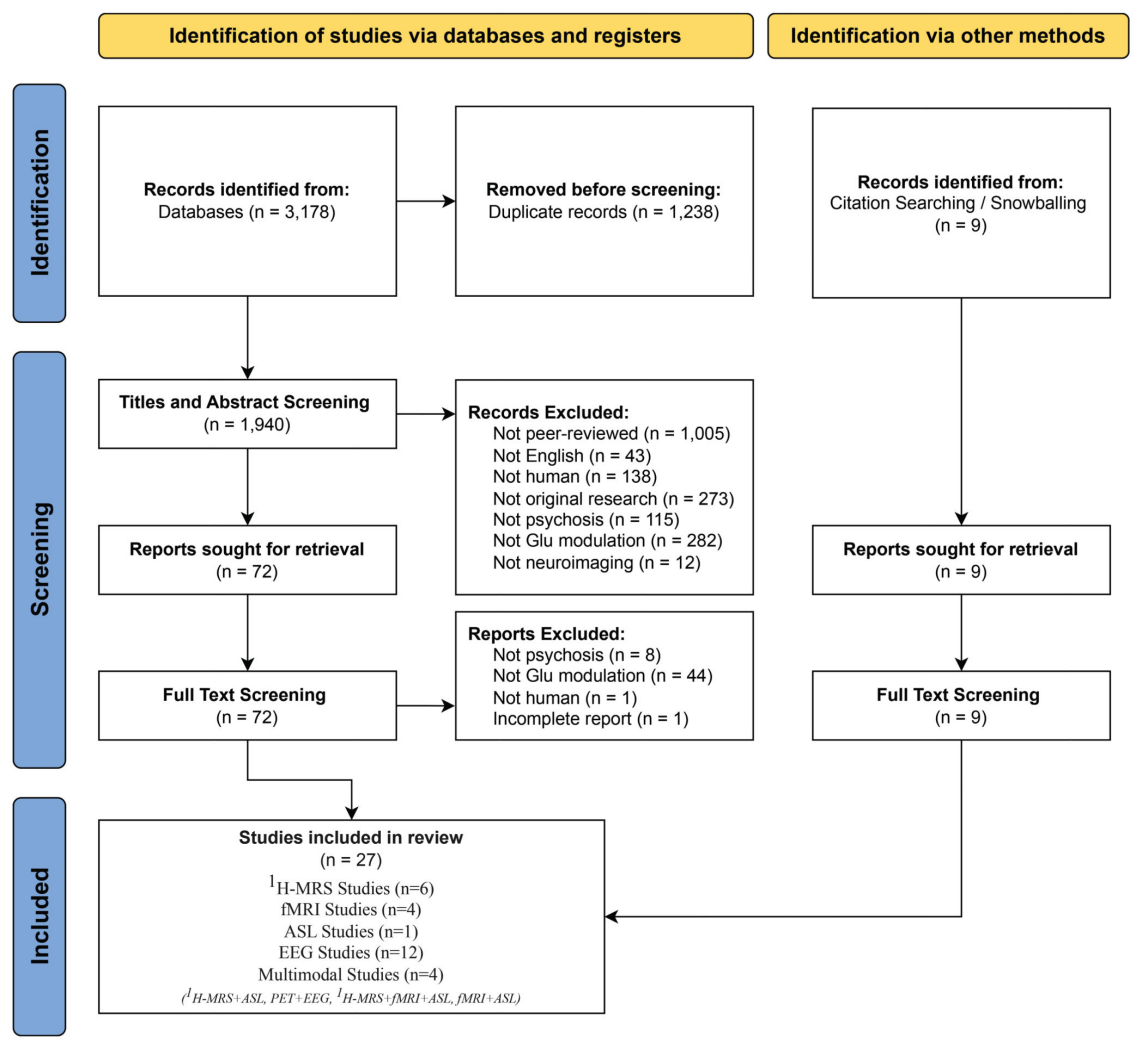
Preferred Reporting Items for Systematic Reviews and Meta-Analyses (PRISMA) flowchart. ASL, arterial spin labeling; EEG, electroencephalography; fMRI, functional magnetic resonance imaging; ^1^H-MRS, proton magnetic resonance spectroscopy.

Second, we summarized neural changes by mechanism of action within pharmacodynamic frameworks (Table 2). Figure 1B illustrates modulators acting on the glutamatergic synapse. Although sex, ethnicity, and illness stage were potential moderators, only one study reported stratified sex analyses and found no sex-by-drug interaction for memantine (43), only one study investigated early psychosis modulation with memantine via EEG measures (44), and no studies stratified their analyses by ethnicity. Full data on demographic characteristics of the included studies are reported in the Supplement.

### Summary of Findings by Neuroimaging Method

#### Proton Magnetic Resonance Spectroscopy

^1^H-MRS can indirectly measure the concentration of Glu, glutamine, and the combination of both (Glx) as markers of glutamatergic functioning. Eight ^1^H-MRS studies investigating the effects of sarcosine, riluzole, *N*-acetylcysteine (NAC), and L-theanine were identified. Sarcosine augmentation of antipsychotic treatment was associated with significant reductions in Glx in the frontal white matter and hippocampus and increases in *N*-acetylaspartate in the dorsolateral PFC and frontal white matter (45–47). L-theanine augmentation was associated with nonsignificant frontal white matter Glx reduction and parietal white matter Glx increases (48). Similarly, riluzole augmentation was associated with a statistically nonsignificant reduction in ACC Glx in individuals with schizophrenia but an increase in Glx in control participants (49), with the interaction reaching statistical significance. Two studies suggested that NAC may reduce Glu concentrations in schizophrenia, with one finding a significant reduction in Glx in the ACC after a single dose (50) and another finding a significant reduction in Glu concentrations in the medial PFC (mPFC) after 8 weeks of treatment (51). However, a third study found no impact of a single dose on Glu concentrations in control participants or patients in the ACC or mPFC (52).

The relationship between ^1^H-MRS measures and clinical outcomes was studied in 4 of the 8 studies. While some reported symptom improvements (45–47), these were not consistently linked to changes in metabolite concentrations (48,51). Notably, half of the studies (49,50,52) did not assess clinical outcomes, likely due to their single-dose or short-duration designs, which may not allow sufficient time for clinical effects to emerge. Overall, despite evidence of neurochemical modulation, the functional relevance of these changes remains unclear due to limited and inconsistent outcome integration. Interestingly, only one study investigated cognitive outcomes (51), but no significant changes were found.

#### Functional Magnetic Resonance Imaging

fMRI indexes neuronal activity indirectly by measuring changes in the blood oxygen level–dependent (BOLD) signal. Six studies were identified, with 4 using resting-state and 2 using task-based fMRI to examine the effects of ketamine, NAC, riluzole, D-cycloserine, and AZD8529.

Resting-state fMRI (rs-fMRI) studies mostly reported functional connectivity, meaning the correlation in BOLD time series between regions of interest. NAC treatment resulted in significantly decreased functional connectivity within the medial frontal cortex and a nonsignificant reduction between the ACC and the frontal pole (53). This indicates a potential normalization of aberrant network connectivity often associated with schizophrenia (54,55). Riluzole had the opposite effect, significantly increasing functional connectivity between the ACC and the anterior PFC in schizophrenia while reducing it in control participants (49).

A related outcome metric is regional homogeneity (ReHo), meaning the synchrony of the BOLD time series among neighboring voxels. Intravenous ketamine administration in patients with schizophrenia with comorbid treatment-resistant depression decreased ReHo within the default mode network, a collection of largely midline cortical regions, and orbitofrontal cortex during the initial weeks of treatment, suggesting a normalization of local brain connectivity (56). However, the effects were not sustained over time (56,57). Notably, the studies did not include an HC group, limiting the ability to determine whether the observed changes reflect a true normalization. Correspondingly, the initial clinical improvements in depressive and psychotic symptoms diminished by the end of the treatment period, indicating that the initial neurobiological changes did not translate into long-term symptomatic relief (56,57).

Task-based fMRI studies indexed BOLD responses (activations) time-locked to task events or inferred latent cognitive variables and provided some evidence that Glu modulators can enhance activation associated with working memory and executive function. Specifically, D-cycloserine increased activation in the temporal gyrus during a verbal fluency task (58), while AZD8529 increased activation in the striatum and ACC during a working memory task (59). In both studies, Positive and Negative Syndrome Scale (PANSS) scores were measured before and after drug administration, revealing a significant improvement in the negative symptom subscale. Notably, these improvements were correlated with increased activation in the left temporal gyrus for D-cycloserine and in the striatum for AZD8529, suggesting that enhanced neural responsiveness to cognitive demands may contribute to the alleviation of negative symptoms (58,59).

Of the 6 fMRI studies, 4 examined associations with symptom (positive and negative) domains only (56–59). While task-based studies showed that improvement in negative symptoms was significantly associated with increased brain activation and synchrony in frontal and temporal cortex (58,59), these symptom improvements were inconsistent or not sustained over time (56,57). Notably, resting-state studies omitted investigating clinical outcomes, likely due to their single dose (49,53) or short duration of intervention (48,49).

#### Arterial Spin Labeling

ASL can quantify regional cerebral blood flow (rCBF) by magnetically labeling inflowing water as an endogenous tracer (60). Four ASL studies were identified, involving isosorbide mononitrate (ISMN), riluzole, AZD8529, and NAC. ISMN treatment over 30 days was associated with significantly increased blood flow in the right thalamus and amygdala after a schedule of 50 mg once daily. This was accompanied by significant clinical and functional improvements as measured by PANSS and Global Assessment of Functioning scores, respectively (61). In contrast, riluzole, AZD8529, and NAC did not show any changes in the rCBF in cortical or basal ganglia structures, and changes in clinical outcomes were not investigated in these studies (49,50,59).

#### Positron Emission Tomography

Our review identified only one PET study (62). This used ^18^F-MK-6577, a ligand for the GlyT1 receptor, and investigated the relationship between dose of PF-03463275 (a Gly-T1 inhibitor) and GlyT1 receptor occupancy. PF-03463275 was shown to bind to GlyT1 receptors in subcortical (pons, midbrain, cerebellum white matter) and cortical (cerebral cortex, thalamus, centrum semiovale) regions postintervention (twice daily for 7 days). Subcortical occupancy was more pronounced than cortical occupancy. The effect of GlyT1 occupancy on positive and negative symptoms was investigated but found to be nonsignificant (62).

#### Electroencephalography

EEG and MEG are direct electromagnetic measures of pooled neuronal activity at the scalp. Although EEG has a relatively poor spatial resolution, it has excellent temporal resolution, making it a complementary tool to other neuroimaging modalities with higher spatial resolution such as fMRI. We identified 13 EEG studies examining the modulatory effects of NAC, glycine, D-serine, memantine, and iclepertin using various EEG paradigms. Most of the tasks used task paradigms targeting low-level sensory (auditory) processes, thought to index predictive processing and oscillatory neuronal entrainment, which in turn are related to cortical excitation-inhibition (E/I) balance: mismatch negativity (MMN), prepulse inhibition (PPI), and auditory steady state response (ASSR).

Schizophrenia is associated with reduced MMN responses, hypothesized to reflect NMDAR hypofunction and linked to negative symptoms (63). Glycine and D-serine enhanced MMN within the auditory cortex, suggesting improved detection of preattentive auditory processing (64–66). However, D-serine was not shown to affect any other component of the event-related potential (ERP), such as N1/100, P200, or contingent negative variation (67), indicating that it may not influence the subsequent stages of auditory processing after sensory detection. In contrast, NAC was shown to increase N1/N100 amplitude in the superior temporal cortex (44), indicating the potential to enhance early sensory processing. Meanwhile, memantine significantly increased MMN amplitude and PPI, especially at higher doses (20 mg) (43,68), and iclepertin had no effect on MMN (69). Similarly, a recent study evaluating the D-amino acid oxidase (DAAO) inhibitor luvadaxistat demonstrated a significant improvement in MMN amplitude at a 50-mg dose in patients with schizophrenia, consistent with enhanced NMDAR function. However, no effect was observed at the higher 500-mg dose, suggesting a dose-dependent response (70).

Studies investigating synaptic plasticity through long-term potentiation (LTP) paradigms yielded mixed results. PF-03463275 enhanced LTP in patients with schizophrenia in a dose-dependent manner, with 40 mg yielding optimal effects, while no significant changes were observed in HC participants. Estimated target occupancy modestly correlated with LTP improvements in an inverted U–shaped pattern, indicating an optimal level of target engagement for synaptic plasticity and the importance of precise dosing (62). In contrast, one study (71) found that D-cycloserine (100 mg single dose) had limited effects on LTP, with an increase in the early C1 component of visual evoked potentials but no significant changes in the later P2 component or overall LTP. This suggests that single dose D-cycloserine may modulate early visual processing without robustly enhancing synaptic plasticity. No clinical outcomes were reported.

Schizophrenia is also associated with reduced gamma power during ASSR, linked to changes in the cortical E/I balance (72). Two studies using treatment with 20 mg of memantine found that it enhanced both the power and intertrial phase coherence (ITPC) of gamma-band responses at 40 Hz (68,73), with stronger effects in younger patients suggesting higher sensitivity to memantine in earlier stages of schizophrenia (68). Conversely, iclepertin did not have a significant effect on gamma-band oscillations (69). Consistent with these findings, luvadaxistat at 50 mg showed a trend toward increased gamma power during ASSR in patients with schizophrenia, although this did not reach conventional significance. No effects were observed at the 500-mg dose (70). The effect of glutamatergic drugs on other frequency bands was also investigated. D-serine modulated neuronal oscillations by reducing alpha and beta power and enhancing theta ITPC in specific contexts (65–67). Notably, theta power increases were only transiently normalizing postdose in one study (67), mirroring the effects observed with high-dose memantine (73).

Of the 13 EEG studies, 3 reported associations with clinical symptoms (64,66,68), and 2 examined cognitive outcomes using the MATRICS Consensus Cognitive Battery (66,69). There is some consistency across the 4 EEG studies reporting clinical associations, particularly in linking enhanced MMN with improvements in negative symptoms following glycine and D-serine treatment (64,66). However, heterogeneity in paradigms, dosing, and outcome measures limits comparisons. Most other studies lacked repeated symptom or cognitive assessments, often due to short, single-dose designs or a mechanistic focus, making broader conclusions about clinical relevance difficult.

### Summary of Findings by Mechanism of Action

Of the 27 studies included, 14 investigated direct gluta-matergic modulators, and 13 investigated indirect modulators; however, the heterogeneity of modalities and compounds makes direct comparisons difficult. The number of studies for direct/indirect modulators was 1/6 for ^1^H-MRS, 4/6 for fMRI, 1/4 for ASL, 1/1 for PET, and 9/14 for EEG studies. With the exception of EEG, this limited number of studies precludes conclusions about direct versus indirect modulator differences.

Within the EEG modality, direct NMDAR modulators such as D-cycloserine (71), glycine (64), D-serine (65–67), and memantine (43,68,73,74) consistently enhanced ERP plasticity and normalized oscillatory synchrony in chronic schizophrenia. By contrast, although NAC, a redox modulator, had promising results in early psychosis, reversing a potential synaptic excitatory deficit in the auditory cortex (44), other indirect modulators such as GlyT1 inhibitors (PF-03463275, iclepertin) and DAAO (luvadaxistat) tended to produce only trend-level changes (62,69,70).

In other modalities, effects did not clearly separate along direct versus indirect modulation. While some direct modulators (NMDAR agonists) boosted regional activation and synchronicity (56–59), others such as mGluR (AZD8529) (59) and AMPAR (L-theanine) (48) showed no effect. Similarly, while some indirect modulators such as NAC and sarcosine yielded inconsistent effects on Glu concentrations (43,45–47,50–52), others such as sodium channel blockers (riluzole) and vasodilators (ISMN) normalized functional connectivity (49) and improved CBF, respectively (61).

## Discussion

We synthesized evidence from 27 studies investigating how Glu modulators affect brain function in schizophrenia across multiple neuroimaging modalities: ^1^H-MRS, fMRI, ASL, PET, and EEG. Overall, various glutamatergic compounds modulate brain function, with some emerging coherence regarding how different classes (direct vs. indirect modulators) affect these measures. However, the relationship between neurobiological changes and clinical or cognitive outcomes remains inconsistent. Despite important progress, mechanistic insights from glutamatergic probes remains underdeveloped.

Our findings can be interpreted using the E/I balance model. Healthy brain function relies on the fine balance between excitatory glutamatergic and inhibitory GABAergic (gamma-aminobutyric acidergic) signaling, essential for neural processing, plasticity, and oscillatory activity across networks (75). Disruptions in this balance, particularly NMDAR hypo-function on parvalbumin-positive GABAergic interneurons (76–78) or impaired astrocytic Glu clearance (79), may disrupt neuronal oscillations, which may in turn disrupt large-scale functional brain networks (80–83), leading to psychiatric conditions (84–87), including psychosis (88–90). These disruptions are observable via ^1^H-MRS, fMRI, and EEG (88,91).

Measuring concentrations of Glu and GABA provides a potential proxy measure for the initial stages of E/I disruption described above. A hyperexcitable state, evidenced by the elevated Glu in the basal ganglia and reduced GABA in the mid- and posterior frontal cortex, has been proposed in early psychosis (92,93). These patterns are less consistent in chronic stages, likely due to regional, clinical, and treatment-related heterogeneity (92–94), although in the subset of individuals with treatment-resistant schizophrenia, there is evidence of raised Glu levels in the medial frontal and midcingulate cortex (92). However, only one non–^1^H-MRS study investigated modulation in early psychosis (44), high-lighting a gap in targeting this stage. In chronic stages, ^1^H-MRS studies found that sarcosine significantly reduced Glu concentrations in the frontal white matter and hippocampus (45–47), while NAC significantly reduced them in the ACC (50) and mPFC (51); however, interpretation is limited by a lack of HC participants. The only treatment-resistant schizophrenia ^1^H-MRS study showed trend-level ACC Glu reduction with riluzole (49), suggesting a normalizing effect given the evidence of raised baseline levels in this population.

Balanced E/I activity enables rhythmic firing patterns that generate neural oscillation, critical for coordinated information processing (95,96). When disrupted, interregional signaling breaks down, driving psychosis and cognitive deficits (95). EEG captures these oscillations, with coherence and ERPs (i.e., MMN) measures emerging as E/I proxies (97,98), found consistently reduced in psychosis, reflecting frontotemporal discoordination and impaired sensory prediction (97,98). Memantine consistently improved these markers in early and chronic psychosis, suggesting a normalization via direct NMDAR modulation (43,68,73,74). Acute glycine, NAC, and luvadaxistat performed similarly (44,64,70); however, in the absence of HC participants, conclusions are limited. GlyT1 blockade with iclepertin had no effect on EEG measures (69), while PF-03463275 effects only occurred at specific doses (62). Convergent changes in other ERPs (i.e., reduced N100) suggest a malleable E/I proxy but need replication.

Oscillatory disruptions may subsequently contribute to macroscale network dysconnectivity, observable using fMRI (83,95,99). Psychosis is often associated with reduced functional connectivity between key brain regions (100,101). The rs-fMRI findings show riluzole’s potential to normalize this dysconnectivity by strengthening ACC-PFC connectivity, possibly driven by the effects on Glu concentrations (49). The effects of other compounds (intravenous ketamine, NAC) on rs-fMRI signals are difficult to interpret without control groups (53,56,57). Furthermore, a lack of task-induced neural activity in fMRI studies may indicate a hyperactive baseline. Therefore, it is possible that the increase in task-related activity observed with D-cycloserine (58) and AZD8529 (59) reflects a reduction in baseline hyperactivity. Similarly, the reduction in CBF following ISMN treatment (61) is consistent with a model in which glutamatergic modulation is able to normalize a hyperactive baseline state, reinforcing its potential therapeutic relevance.

Our synthesis suggests that direct NMDAR modulators may be associated with relatively consistent effects across neuroimaging measures, in contrast to less reliable effects of indirect modulators. Several indirect modulators (iclepertin, bitopertin, DAO inhibitors) showed early clinical promise but failed in later-phase clinical trials (39,102,103). The use of neuroimaging measures to confirm target engagement and dose–response relationships could potentially help to reduce the risk of compound development in future. Similarly, neuroimaging could be used to probe why some compounds succeed, as in the case of evenamide, which has recently shown phase 3 efficacy in treatment-resistant schizophrenia (104). Neuroimaging could play a key role in identifying optimal biological dosing by capturing intermediate brain response patterns. A systematic incorporation of neuroimaging markers during early-phase trials could improve trial design and prevent late-stage failure.

### Strength and Limitations

By integrating evidence from ^1^H-MRS, fMRI, ASL, PET, and EEG, the review provides a multimodal perspective on glutamatergic modulation. However, several limitations merit consideration. First, small sample sizes, heterogeneity in intervention protocols (e.g., compound type, dose, duration), and regional variability in glutamatergic abnormalities limit comparability and preclude meta-analysis. Second, neuroimaging measures only measure Glu signaling indirectly, limiting mechanistic insights. Third, the frequent absence of HC groups often precludes mechanistic interpretation, and omission of clinical and cognitive end points undermines translational relevance. Fourth, all but one study focused on chronic psychosis, leaving modulation in early psychosis largely unexplored, although it may be the patient group most responsive to intervention. Finally, we observed a lack of demographic stratification. Only 3 studies reported sex as a covariate (48,65,71) and one as a moderator (43). This limits insight into sex-specific treatment effects despite evidence that glutamatergic function (105,106) and cognition (107) differ by sex. Similarly, ethnicity data were inconsistently reported, with predominantly White samples restricting generalizability and obscuring population-specific responses (108).

### Future Directions

Progress toward effective, personalized glutamatergic interventions in psychosis will require a shift in study design and scope. Future research could prioritize early-stage and prodromal populations, in which glutamatergic dysfunction may be more therapeutically modifiable. To support generalizability and uncover population-specific effects, increased efforts to recruit larger and more diverse samples should be made. In this context, embedding neuroimaging in early-phase drug trials can potentially help confirm target engagement, identify dose-response relationships, and clarify mechanisms behind both treatment success and failure. Finally, new biomarkers may also help advance these insights, with novel PET tracers or metrics based around functional autocorrelation (e.g., Hurst exponent) showing promise (86,109).

## Conclusions

This systematic review highlights the capacity of glutamatergic modulators to alter brain Glu concentrations, functional connectivity, and electrophysiological indices in psychosis. However, these neuroimaging improvements have not reliably translated into sustained clinical benefits, especially for negative and cognitive symptoms. Larger, more standardized, and multimodal studies including longer follow-up are needed to refine our understanding of which agents, doses, and patient subgroups may realize enduring therapeutic gains from glutamatergic interventions. Importantly, by bridging the gap between neuroimaging biomarkers and clinical outcomes, future research can pave the way toward more personalized and effective therapeutic strategies for the management of psychosis.

## Supplementary Material

Supplementary material cited in this article is available online at https://doi.org/10.1016/j.bpsc.2025.07.004.

Supplementary material

## Figures and Tables

**Table 1:** Summary of the ^1^H-MRS, fMRI, ASL, PET, and EEG Studies

Modulator Mechanism	Author, Year	Glu Modulator	Study Design	Scz:HC/ PBO, *n*	Outcome Measured	Brain Location	Key Changes	Clinical Outcomes
^1^H-MRS Studies								
Direct	Ota *et al.,* 2015 (48)	L-theanine, 250 mg, OD, 2 months	Chronic Scz. Open-label, pre-post, single-group, Scz only vs. HC only at BL B_0_ = 1.5T	17:0	Metabolite changes	Left middle frontal white matter	All decreased except alpha-GPC + PCho, which remained similar	Significant improvement in PANSS total and PSQl pre- and posttreatment but no significant correlations between Glx and clinical outcomes. Higher BL Glx levels showed a significant reduction in Glx after treatment.
						Left inferior parietal white matter	⟷ between HC and Scz at BL ⟷ alpha-GPC + PCho and NAA ↑ Glx ↓ ml	
Indirect	Strzelecki *et al.,* 2015 (45–47)	Sarcosine, 2 g,OD, 2 months	Chronic Scz. Double-blinded, RCT PBO, pre-post, parallel, Scz only B_0_ = 1.5T	25:25	Metabolite changes	Left frontal white matter	↓ Glx: Cr^a^, Glx: Cho^a^, NAA: Cho ↑ NAA: Cr^a^	Significant improvement in PANSS negative, general, and total.
						Left dlPFC	↑ NAA: Cr^a^, NAA: Cho^a^, mI:Cr^a^, ml: Cho^a^	
						Left hippocampus	↓ Glx: Cr^a^, Glx: Cho^a^	
	McQueen *et al.,* 2018 (50)	NAC, 2400 mg, single dose	Chronic Scz. Double-blinded, RCT PBO, pre-post, crossover, Scz only B_0_ = 3T; CSFc	19:0	Metabolite changes	ACC	↓ Glx: Cr^a^	No clinical or cognitive outcomes reported pre-post intervention.
						Caudate nucleus	↑ Glu, Glx, NAA, Cho, ml, Cr, Glx:Cr ⟷ Cho: Cr ↓ Glx: Cr, NAA: Cr, ml: Cr	
	Pillinger *et al.,* 2019(49)	Riluzole, 50 mg, BD, 2 days	TRS. Open-label, within-subjects, PBO, pre-post, parallel vs. HC B_0_ = 3T; CSFc	19:18	Metabolite changes	ACC	↑ Glx, Glx: Cr, NAA: Cr, Glx: Cr in HC ↓ Glx, Glx: Cr and ↑ Glx: Cr, NAA: Cr Scz	PANSS only at BL and ↑Glx significantly correlated with ↑PANSS No other statistically significant findings
	Girgis *et al.*, 2019 (52)	NAC, 2400 mg, single dose	Chronic Scz. Double-blinded, RCT PBO, pre-post, parallel vs. HC B_0_ = 3T; CSFc	19:20	Metabolite changes	ACC	↓ Glx, ↓ Glu and ⟷ GSH in Scz vs. HC at BL ↑ Glx and ↑ GSH post NAC in Scz ↓ Glx and ↑ GSH in HC post NAC GSH ↑ in HC > Scz	No clinical or cognitive outcomes reported pre-post intervention.
						Medial PFC	↓ Glx and ⟷ Glu in Scz vs. HC at BL ↑ Glx, ↑ Glu post NAC in Scz ↓ Glx and ↓ Glu in HC post NAC	
	Yang *et al.*, 2022 (51)	NAC, 1200 mg, BD, 8 weeks	Chronic Scz. Double-blinded, RCT PBO, pre-post, parallel Scz only B_0_ = 3T	18:16	Metabolite changes	Left dlPFC	⟷ GSH: Cr ⟷ Glx: Cr ⟷ GSH: GSSG	No statistically significant correlations with PANSS, CAINS, or MCCB scores
						Medial PFC	↑ GSH: Cr^a^ ↓.Glx: Cr (turns ^a^ in sensitivity analysis) ⟷ GSH: GSSG	
fMRI Studies								
Direct	Ye *et al.,* 2019 (56)	Ketamine, IV, 0.5 mg/kg weekly, 28 days	TRS. Open-label, single-group, pre and post, Scz only B_0_ = 3T, rs-fMRI	12:0	ReHo values at BL, days 7, 14, 21, and 28	DMN and OFC	↑ ReHo^a^ at days 7, 14, and 21 but not sustained at day 28 in mPFC, ACC, PCC, precuneus, angular gyrus, and OFC postintervention	CDSS and PANSS were measured weekly and showed a significant improvement in CDSS scores from day 7 to day 14 but decreased to BL levels by day 28.
	Zhuo *et al.,* 2020 (57)	Ketamine, IV, 0.5 mg/kg weekly, 28 days	TRS. Open-label follow-up study of Ye *et al*. (56) Single-group, pre and post, Scz only B_0_ = 3T, rs-fMRI	12:0	ReHo values at BL (day 58), days 65, 72, and 79	DMN and OFC	↑ ReHo^a^ at day 65 in temporal, frontal, and parietal lobes, which continue to remain ↑ at 72, but lower *t*values	CDSS and PANSS were measured weekly, but no significant differences compared with the BL.
	Yurgelun *et al.,* 2005 (58)	D-cycloserine, 20 mg, OD, 2 months	Chronic Scz. Double-blinded, RCT PBO, pre-post, parallel, Scz only B_0_ = 1.5T, t-fMRI (block design)	6:6	ROI activation	Superior temporal and anterior cingulate bilateral gyri	⟷ activation in anterior cingulate bilaterally ↑ activation in left temporal gyrus^a^ and ⟷ activation in right temporal gyrus in the intervention group	PANSS was measured before and after and found a significant improvement in negative subscale associated with left temporal gyrus activation.
	Wolf *et al.,* 2022 (59)	AZD8529, 80 mg, OD, 3 days	Chronic Scz. Double-blinded, RCT PBO, pre-post, crossover, Scz only B_0_ = 3T; t-fMRI (block design)	26:0	ROI activation and cognitive performance (via d’ measure—task performance)	PFC (dLPFC and ACC) and striatum	↑^a^ activation in ACC and striatum and ↔ in DLPFC in the intervention group ⟷ effects on the d’ measure	PANSS was measured before and after and found a significant improvement in negative subscale associated with striatal activation.
Indirect	McQueen, 2020 (53)	NAC, 2400 mg, single dose, orally, 1 day	Chronic Scz. Double-blinded, RCT PBO, pre-post, crossover, Scz only B_0_ = 3T; rs-fMRI.	19:0	rs-FC	DMN and SN	↓ rs-FC^a^ in DMN (mPFC and mFG) and in SN (ACC and the frontal pole) postintervention	No clinical or cognitive outcomes reported pre-post intervention.
	Pillinger *et al.,* 2019 (49)	Riluzole, 50 mg, BD, 2 days	TRS. Open-label, within-subjects, PBO, pre-post, parallel vs. HC B_0_ = 3T; rs-fMRI	19:18	rs-FC	ACC seed to whole brain	↓ rs-FC ACC-aPFC^a^ in Scz group vs. HC at BL ↑ rs-FC ACC-aPFC^a^ in Scz group and ↔ in HC postintervention	PANSS was measured at BL only, with no significant correlations found between PANSS scores and frontal connectivity.
ASL Studies								
Direct	Wolf *et al.,* 2022 (59)	AZD8529, 80 mg, OD, 3 days	Chronic Scz. Double-blinded, RCT PBO, pre-post, crossover, Scz only B_0_ = 3T	26:0	rCBF	PFC (dLPFC and ACC) and striatum	⟷ no significant difference between intervention and PBO, no trends presented	No correlation between rCBF and clinical outcomes presented.
Indirect	Guimaraes *et al.,* 2021 (61)	ISMN, 50 mg, OD, 30 days	Chronic Scz. Double-blinded, RCT PBO, pre-post, crossover, Scz only B_0_ = 3T	24:0	rCBF	Right thalamus, right angular gyrus, right amygdala	Between-group ↓ CBF in right thalamus^a^(intervention vs. BL) ↓ CBF in right angular gyrus^a^ (intervention vs. BL) Within-group ↑ CBF in left hippocampus ^a^ (PBO) ↓ CBF in right thalamus^a^(intervention) ↓ CBF in right amygdala ^a^(intervention) ↑ CBF in left angular gyrus^a^ (intervention) ↓ CBF in right dlPFC (intervention) ↓ CBF in right supramarginal gyrus ^a^(intervention) Post hoc between-group ↑ CBF in right thalamus ^a^(intervention vs. BL) ↑ CBF in right amygdala ^a^(intervention vs. BL)	PANSS total score, positive symptoms, and GAF improved significantly in week 3-4 of ISMN treatment.
	Pillinger *et al.,* 2019 (49)	Riluzole, 50 mg, BD, 2 days	TRS. Open-label, within-subjects, PBO, pre-post, parallel vs. HC B_0_ = 3T	19:18	rCBF	ACC, whole brain	⟷ no significant group × time effects between groups	PANSS was measured at BL only, with no significant correlations found between PANSS scores and rCBF.
	McQueen *et al.,* 2018 (50)	NAC, 2400 mg, single dose	Chronic Scz. Double-blinded, RCT PBO, pre-post, crossover, Scz only B_0_ = 3T	19:0	rCBF	ACC, caudate nucleus	⟷ no significant difference between intervention and PBO	No correlation between rCBF and clinical outcomes.
PET Studies								
Indirect	D’Souza *et al.,* 2018 (62)	PF-03463275, 10, 20, 40 mg, BD, 1 week	Chronic Scz. Double-blinded, RCT, pre-post, parallel vs. HC (substudy 1)	9:23	GlyT1 receptor occupancy	13 ROIs: cortical and subcortical regions	↑ GlyT1 occupancy in both subcortical regions: pons, midbrain, cerebellum white matter ^a^, and cortical regions (cerebral cortex, thalamus, centrum semiovale) *’* in the intervention group GlyT1 subcortical occupancy > GlyT1 cortical occupancy ↓ VT across all brain regions with increasing dose ^a^(dose-response)	No significant correlations were reported between GlyT1 occupancy and PANSS scores.
	D’Souza *et al.,* 2018 (62)	PF-03463275, 60 mg, BD, 1 week	Chronic Scz. Double-blinded, RCT, pre-post, crossover, Scz only (substudy 2)	10:0	GlyT1 receptor occupancy	13 ROIs: cortical and subcortical regions	↑ GlyT1 occupancy at 60 mg (vs. PBO)	No significant correlations were reported between GlyT1 occupancy and PANSS scores.
EEG Studies								
Direct	Forsyth *et al.,* 2017 (71)	D-cycloserine, 100 mg, single dose	Chronic Scz. Double-blinded PBO RCT, pre-post, parallel Scz only. Standard checkboard visual stimulus	24:21	Performance on the n-back working memory, weather prediction, and information integration task LTP (negative C1 and positive P2 components of VEP amplitudes)	Primary visual cortex for the LTP	↑ in C1^a^ but ⟷ in P2 postintervention ⟷ LTP (either C1 or P2) postintervention	No clinical or cognitive outcomes reported pre-post intervention.
	Greenwood *et al.,* 2018 (64)	Glycine, 0.2 g/kg (acute - once) or 0.6 g/kg/day (chronic—6 weeks)	Chronic Scz. Double-blinded PBO, RCT, pre-post, parallel, Scz only (HC BL comparison only) 19 electrodes, sampling rate: 250 Hz; MMN paradigm	12:10	MMN amplitude and frequency of the ERP	Auditory cortex	↓ MMN amplitude ^a^ and ⟷ frequency in Scz vs. HC at BL ⟷ MMN post overall glycine (acute 1 chronic administration) ⟷ MMN chronic glycine administration on its own ↑ in MMN amplitude in the acute glycine administration group^a^	Chronic glycine significantly reduces PANSS total, negative, and general scores but no changes in positive PANSS and functional (WSAS) outcomes. Trend-level improvement in CDRS.
	Kantrowitz *et al.,* 2018 (66)	D-serine, 4 g, OD, 6 weeks	Chronic Scz. Double-blinded PBO, RCT, pre-post, crossover, Scz only 65 electrodes, sampling rate: 512 Hz Auditory oddball paradigm	11:0	MMN amplitude, frequency, and duration (for deviants) and N1 amplitude (standard stimuli) of the ERP Time-frequency measures (power and coherence) in alpha and theta bands	Auditory cortex	↑ MMN frequencyz ⟷ MMN amplitude and duration; ⟷ N1 ↑evoked power in α-band for N1^a^ ↓ evoked power in α-band ^a^ and ↑ evoked single trial power in θ band for MMN ^a^ Coherence measures showed ↑ trends in phase consistency	PANSS total and negative scores show a significant improvement in the D-serine group. No significant change in MCCB composite scores. Frequency MMN amplitude significantly correlated with PANSS improvement.
	Molina *et al., 2020* (74)	Memantine, 10 or 20 mg, single dose	Chronic Scz. Double-blinded PBO RCT, pre-post, crossover, Scz only (HC BL comparison) 64 electrodes, sampling rate: 2048 Hz downsampled to 512 Hz Auditory oddball paradigm	36:31	1/f-like aperiodic slope and oscillatory power in the theta, alpha, gamma bands for spectral decomposition MMN amplitude and PPI	Global	The 1/f aperiodic slope is ↑ (steeper) in Scz (PBO) vs. HC; ↓ (flattened) in Scz (MEM 20 mg) vs. HC ^a^ and ⟷ in Scz (MEM 10 mg) vs. HC at BL ↑ Θ and α and ↓ γ power in Scz vs. HC ^a^ at BL ⟷ across power spectrum and in the 1/f aperiodic slope post-MEM 10 mg ↓ in α ^a^, ↑ γ ^a^, ⟷ Θ power and ↓ 1/f aperiodic slope in Scz (MEM 20 mg) vs. HC^a^	No clinical or cognitive outcomes reported pre-post intervention.
	Govani *et al.,* 2023 (67)	D-serine, 100 mg/ kg weekly for 3 weeks + auditory remediation therapy	Chronic Scz. Double-blinded PBO, RCT, pre-post, parallel Scz only 64 electrodes, sampling rate: 500 Hz, MMN (pitch discrimination between S1 and S2) paradigm	12:9	N1, P2, and CNV amplitude and latency of the ERP for both S1 and S2 Time-frequency measures (power and coherence) in beta and theta bands across 3 intervals: 0-200 ms (early response S1 and S2), 400-100 ms (retention post S1) and 200-500 ms (motor preparation post-S2)	Auditory cortex Premotor and frontal cortex	⟷ in the N1, P2, and CNV post D-serine ↑ in Θ power initially (early response), but ⟷ Θ power and ⟷ Θ — ITC post D-serine between groups ↓ in β power leading to ↑ β—ERD post D-serine in the retention and motor preparation interval^a^	No clinical or cognitive outcomes reported pre-post intervention.
	Sehatpour *et al.,* 2023 (65)	D-serine, 80, 100, 120mg/kg, single doses, weekly, 3 weeks	Chronic Scz. Double-blinded PBO RCT, pre-post, parallel Scz only 64 electrodes; sampling rate: 500 Hz AudRem (pitch discrimination between S1 and S2) and MMN paradigms	36:9	“Plasticity improvement” (%Δf in pitch discrimination threshold) MMN pitch amplitude deviant and P2b amplitude of the ERP Time-frequency measures: Θ— ITC and β—ERD specifically	Auditory cortex, frontoparietal networks	↑ %Δf within treatment groups ^a^ and between treatment and PBO groups post-D-serine^a^ ↑ %Δf in 80 and 100 mg and ↓ %Δf in 120 mg when compared with each other ↑ MMN pitch^a^ at theW2 but ↓ by W3 for D-serine (100 mg) and no significant trends for D-serine (80 and 120 mg) or ↑ P2b post-D-serine ↑ β—ERD post D-serine 100 mg ^a^ and t in Θ— ITC	No clinical or cognitive outcomes reported pre-post intervention.
	Swerdlow *et al.,* 2024 (73)	Memantine, 20 mg, single dose	Chronic Scz. Double-blinded PBO, RCT, pre-post, crossover Scz vs. HC 64 electrodes; sampling rate: 1024 Hz; 40 Hz ASSR and 80 Hz SSHR paradigms	28:25	Time-frequency measures including evoked power, ITC, and harmonic ratio F1:F2	Auditory cortex	↓ evoked gamma power ^a^ ↓ ITC at F1 and F2 ^a^ and ↓ F1:F2 ^a^in Scz > HC at BL ↑ in evoked gamma power at 1F in both HC and Scz groups^a^, but ⟷ in EP at 2F post-MEM ↑ in ITC at 1F in both HC and Scz groups ^a^, but ⟷ in ITC at 2F post-MEM. ↓ F1:F2 in Scz vs. HC	No clinical or cognitive outcomes reported pre-post intervention.
	Swerdlow *et al.,* 2016 (43)	Memantine, 10, 20 mg, single dose	Chronic Scz. Double-blinded PBO, pseudo—RCT, pre-post, crossover, Scz vs. HC 64 electrodes; sampling rate 2048 Hz, downsampled at 512 Hz PPI startle and auditory oddball MMN paradigm	41:43	PPI MMN amplitude Startle magnitude and habituation	Frontal and temporal cortex for PPI and MMN and subcortical regions (ventral striatum, pallidum, and pontine tegmentum) for PPI	⟷ BL PPI between Scz and HC ↓ PPI post-MEM 10 mg in Scz vs. HC & ↑ PPI post-MEM 20 mg in Scz vs. HC^a^ ↓ BL MMN in Scz vs. HC^a^ ⟷ MMN post-MEM 10 mg and ↑ MMN in both Scz and HC post-MEM 20 mg ^a^ No group differences Younger participants showed a larger MMN enhancing effect^a^ BL startle. ↓ Startle in Scz vs. HC and ⟷ post-MEM 10 or 20 mg in either group	No clinical or cognitive outcomes reported pre-post intervention.
	Light *et al.,* 2017 ~ (68)	Memantine, 20 mg, 2 days	Chronic Scz. Double-blinded PBO, RCT, pre-post, crossover Scz vs. HC 64 electrodes; sampling rate 2048 Hz ASSR paradigm	18:14	γEP and γPL	Frontal and temporal cortex	↓ γEP and γPL in Scz vs. HC at BL measurements^a^ ↑ γEP and γPL in Scz and HC post-MEM 20 mg, but no significant differences between the groups	A Symptom Rating Scale was used at multiple time points (BL, 30, 90, 150, 200, and 230 minutes post-pill). No significant changes post-MEM.
Indirect	D’Souza *et al.,* 2018 (62)	PF-03463275, 60 mg BD, + week	Chronic Scz. Double-blinded, RCT, pre-post, crossover, Scz only Substudy 2: 2-stimulus visual odd ball task + block of high-frequency photic stimulation	10:0	LTP (N100 negative deflection change of VEP amplitudes) ETO (target occupancy) and LTP relationship	Primary visual cortex (implied from VEPs)	⟷ VEP amplitudes in Scz groups at BL ⟷ VEP amplitudes in Scz groups post-60 mg (inverted U dose shape at 60 mg) Combined substudy 1 + substudy 2 for ETO-LTP relationship and found a quadratic relationship, but it was nonsignificant	No clinical or cognitive outcomes reported pre-post intervention.
	D’Souza *et al.,* 2018 (62)	PF-03463275, 10, 20, 40 mg, BD, 1 week	Chronic Scz. Double-blinded, RCT, pre-post, Parallel vs. HC Substudy 1: two-stimulus visual odd ball task 1 block of high frequency photic stimulation	9:23	LTP (N100 negative deflection change of VEP amplitudes) ETO (target occupancy) and LTP relationship	Primary visual cortex (implied from VEPs)	⟷ VEP amplitudes in Scz and HC at BL ↑ VEP amplitudes in Scz group at 40 mg ^a^ but not at 10 and 20 mg post-intervention ⟷ VEP amplitude in HC across dosages postintervention When ETO ↑, LTP moderately ↑, but borderline significant	No clinical or cognitive outcomes reported pre-post intervention.
	Schultheis *et al.,* 2022 (69)	Iclepertin (BI 425809), 2, 5, 10, 25 mg, OD, 12 weeks	Chronic Scz. Double-blinded PBO RCT, pre-post, parallel, Scz only 30 electrodes, sampling rate: 512 Hz MMN and ASSR paradigms and resting-state EEG	59:20 (14; 10; 20; 15 vs. 20)	MMN amplitude (frequency, duration, and double deviants) ASSR (phase locking factor, evoked and induced power) Resting-state gamma power (absolute and relative)	Auditory cortex	⟷ in the MMN, ASSR, and resting-state gamma power post-iclepertin	MCCB score improved nonsignificantly in the intervention group vs. PBO.
	O‘Donnell *et al.,* 2023 (70)	Luvadaxitat, 50 or 500 mg, OD, 8 days	Chronic Scz. Double-blinded, PBO, RCT, pre-post, crossover, Scz only 64 electrodes; sampling rate 1 kHz; MMN (oddball), ASSR (40-Hz gamma) and active oddball task (P300 wave) paradigms	31:0	MMN amplitude ASSR γ band power Evoked potential P300 amplitude EBC measured as average percentage of responses	Cerebellum and cerebellar circuitry specified Not specifically stated, but frontal and temporal cortex implied	↑ EBC for 50 mg and ⟷ for 500 mg ↑ MMN amplitude ^a^ for 50 mg and ⟷ for 500 mg ↑ γ power for 50 mg and ⟷ for 500 mg ↓ P300 amplitude with 50 mg (vs. PBO t), and ↑ with 500 mg (but PBO ↑ even more)	The BACS was used, but data were deemed uninterpretable due to learning effect and BL variability.
	Retsa *et al.,* 2018 (44)	NAC, 2700 mg, OD, 6 months	Early psychosis. Double-blinded PBO RCT, pre-post, parallel, Scz only (HC BL comparison only) 64 electrodes, sampling rate: 1024 Hz; auditory oddball paradigm	8:7	N100 amplitude of the AEP (ERP) and source activity	Left and right posterior-superior temporal cortex	↓, N100 amplitude in Scz vs. HC at BL^a^ ↑ N100 amplitude^a^ at multiple time windows and ↑ source activity in left temporal cortex^a^ in the Scz group (NAC vs. PBO)	No clinical or cognitive outcomes reported pre-post intervention.

B_0_ = magnetic field strength measured in tesla (T). Arrows denote the trends (↑ = increase, ↓ = decrease, ↔ no change/difference). N1/100 and P2 are ERP components that reflect auditory processing. Oscillation band ranges: θ: 4–8 Hz, α: 8–12 Hz, β: 13–30 Hz, γ: 30–100 Hz. For MRS: metabolites of interest were Glx, Glu, Cr, Cho (including PCho and GPC), NAA, mI, GSH, GSSG and their ratios for, e.g., Glx:Cr. 1F/2F are the fundamental frequency (F1) and second harmonic (F2) in ASSR. ACC, anterior cingulate cortex; AEP, auditory evoked potential; aPFC, anterior PFC; ASL, arterial spin labeling; ASSR, auditory steady state response; AudRem, auditory remidiation; BACS, Brief Assessment of Cognition in Schizophrenia; BD, twice daily; BL, baseline; CAINS, Clinical Assessment Interview for Negative Symptoms; CBF, cerebral blood flow; CDSS, Calgary Depression Scale for Schizophrenia; Cho, choline; CNV, contingent negative variation; Cr, creatine; CSFc, cerebrospinal fluid correction; dLPFC, dorsolateral PFC; DMN, default mode network; EBC, eyeblink conditioning; EEG, electroencephalography; EP, evoked power; ERD, event-related desynchronization; ERP, event-related potential; ETO, estimated target occupancy; fMRI, functional magnetic resonance imaging; GAF, Global Assessment of Functioning; Glu, glutamate; Glx, glutamate 1 glutamine; GlyT1, glycine transporter type 1; GPC, glycerophosphocholine; GSH, glutathione; GSSG, glutathione disulfide; HC, healthy control participants; ISMN, isosorbide mononitrate; ITC, intertrial coherence; IV, intravenous; LTP, long-term potentiation; MCCB, MATRICS Consensus Cognitive Battery; MEM, memantine; mFG, medial frontal gyrus; mI, myo-inositol; MMN, mismatch negativity; mPFC, medial PFC; MRS, magnetic resonance spectroscopy; NAA, *N*-acetylaspartate; NAC, *N*-acetylcysteine; OD, once daily; OFC, orbitofrontal cortex; PANSS, Positive and Negative Syndrome Scale; PBO, placebo; PCC, posterior cingulate cortex; PCho, phosphocholine; PET, positron emission tomography; PFC, prefrontal cortex; PPI, prepulse inhibition; PSQI, Pittsburgh Sleep Quality Index; rCBF, regional cerebral blood flow; ReHo, regional homogeneity; ROI, region of interest; rs-FC, resting-state functional connectivity; SCZ, schizophrenia; SN, salience network; t-fMRI, task fMRI; TRS, treatment-resistant schizophrenia; VEP, visual evoked potential; VT, total distribution volume in PET imaging; WSAS, Work and Social Adjustment Scale.

a Statistically significant, *p*,.05.

**Table 2:** Overview of Glutamate Modulators Reviewed and the Articles Included With Their Neuroimaging Methods of Choice

	Modulator Mechanisms			Articles Identified per Modulator	
	Compound	Mechanism	Location	No. of Articles, Total Sample Size	Neuroimaging Modalities
Direct	Ketamine	NMDAR antagonist at ion channel pore	Postsynaptic	2 articles, 12	fMRI
	D-cycloserine	NMDAR agonist at GBS	Synaptic cleft	2 articles, 57	fMRI, EEG
	D-serine	NMDAR agonist at GBS	Synaptic cleft	3 articles, 77	EEG
	Glycine	NMDAR agonist at GBS	Synaptic cleft	1 article, 22	EEG
	Memantine	Partial noncompetitive NMDAR antagonist at ion channel pore	Postsynaptic	4 articles, 236	EEG
	AZD8529	mGlu_2_ positive allosteric modulator	Presynaptic	1 article, 26	fMRI
	L-theanine	AMPAR and NMDAR antagonist	Postsynaptic	1 article, 39	MRS
Indirect	Sarcosine	GlyT1 inhibitor	Synaptic cleft	3 articles, 50	MRS
	Iclepertin	GlyT1 inhibitor	Synaptic cleft	1 article, 77	EEG
	PF-03463275	GlyT1 inhibitor	Synaptic cleft	1 article, 32	PET
	Riluzole	VGSC blockage	Presynaptic	1 article, 37	MRS, fMRI, ASL
	NAC	Xc^−^ system enhancement	Synaptic cleft	5 articles, 121	MRS, EEG, ASL, fMRI
	ISMN	NO donor—smooth muscles vasodilator-increases CBF	Whole brain	1 article, 24	ASL
	Luvadaxistat	DAAO inhibitor—increases D-serine availability	Astrocytes	1 article, 31	EEG

AMPAR, AMPA receptor; ASL, arterial spin labeling; CBF, cerebral blood flow; DAAO, D-amino acid oxidase; EEG, electroencephalography; fMRI, functional magnetic resonance imaging; GBS, glycine binding site; GlyT1, glycine transporter type 1; ISMN, isosorbide mononitrate; MRS, magnetic resonance spectroscopy; NAC, *N*-acetylcysteine; NMDAR, NMDA receptor; NO, nitric oxide; PET, positron emission tomography; VGSC, voltage-gated sodium channel; Xc^−^, cysteine/glutamate antiporter.
