# Supplementary material for: Glutamatergic Modulation of Brain Function in Psychosis: A Systematic Review of Neuroimaging Studies
Source: Biol Psychiatry Cogn Neurosci Neuroimaging. Author manuscript; Available in PMC 2026 Apr 27. (PMC7619035; doi:10.1016/j.bpsc.2025.07.004)
Supplement: Supplementary material [file EMS213389-supplement-Supplementary_material.docx]

**SUPPLEMENTARY INFORMATION**

**Glutamatergic Modulation of Brain Function in Psychosis: A Systematic Review of Neuroimaging Studies**

Varvari *et al.*

**Supplementary material**

**Section 1:** Search Strategy String

**TEXT Box 1**. ***Search Strategy Employed***

(psychosis OR psych* OR schizophrenia OR schizop*) AND (human studies) and (magnetic resonance imaging OR MRI OR functional magnetic resonance imaging OR fMRI OR positron emission tomography OR PET OR magnetic resonance spectroscopy OR MRS OR EEG OR electroencephalography OR magnetoencephalography OR MEG OR event related potential OR ERP OR voxel-based morphometry OR VBM OR diffusor tensor imaging OR DTI OR SPECT or Positron Emission Tomography OR PET OR Computer Tomography OR CT) AND (glutamat* modulat* OR NMDA* OR lamotrigine OR memantine OR ketamine OR d-serine OR d-cycloserine OR glycine OR mGlu* OR pomaglumetad OR Basmisanil OR mavoglurant OR glycine transporter inhibitor OR sarcosine OR biopterin OR glutamate release inhibit* OR Riluzole).

**Section 2:** Demographic Table as reported per study

| **Study** | **Patients Gender** | | **Healthy Controls** | | **Ethnicity Report** |
| --- | --- | --- | --- | --- | --- |
|  | Female | Male | Female | Male | HC:PT |
| *Sehatpour et. al. 2023* | 7 | 29 | 2 | 7 | *not stated* |
| *Govani et. al. 2023* | 6 | 6 | 7 | 2 | *not stated* |
| Yang et al., 2022 | 3 | 12 | 4 | 11 | *not stated* |
| Wolf et al., 2022 | 10 | 16 | *no healthy control group* | | *not stated* |
| *Schultheis et al., 2022* | 6 | 14 | 12 | 47 | 64.6% White |
| *Guimaraes et al., 2021* | 2 | 18 | *no healthy control group* | | *not stated* |
| *Kantrowitz et al., 2018* | 1 | 10 | *no healthy control group* | | *not stated* |
| *McQueen et al.,2018a,b.* | 3 | 17 | *no healthy control group* | | *not stated* |
| *Retsa et al. 2018, a,b* | 2 | 13 | 3 | 15 | *not stated* |
| *Strzelecki et al., 2015a,b,c* | 14 | 11 | 12 | 13 | *not stated* |
| *D'Souza et al., 2018 substudy1* | 1 | 8 | 4 | 19 | *10:5 AA*  *10:3 Caucasian*  *3:0 Asian*  *0:1 Other* |
| *D'Souza et al., 2018 substudy2* | 2 | 8 | *no healthy control group* | | *4 AA*  *3 Caucasia*  *1 Asian*  *2 Other* |
| *Yurgelun-Todd et al., 2015* | 1 | 5 | 1 | 5 | *not stated* |
| *Forsyth et al., 2017* | 3 | 21 | 5 | 16 | *not stated* |
| *Miho Ota et al., 2015* | 8 | 9 | *no healthy control group* | | *not stated* |
| *McQueen et al., 2020* | 3 | 14 | *no healthy control group* | | *not stated* |
| *Ye et al., 2019* | 5 | 7 | *no healthy control group* | | *not stated* |
| *Zhuo et. al., 2020* | 5 | 7 | *no healthy control group* | | *not stated* |
| *Swerdlow et. al., 2016* | 12 | 29 | 9 | 34 | *17:15 Caucasian 7:6 Hispanic*  *14:5 Asian*  *4:10 AA*  *1:5 Other* |
| *Swerdlow et. al., 2024* | 9 | 19 | 13 | 12 | *32%:35.7% White* |
| *Molina et al., 2020* | 12 | 24 | 24 | 7 | *not stated* |
| *Greenwood et al., 2018* | 12 | 10 | 12 | 9 | *not stated* |
| *Pillinger et al., 2019* | 3 | 16 | 3 | 15 | *not stated* |
| *Girgis et al., 2019* | 5 | 14 | 6 | 14 | *24 AA*  *8 Caucasian*  *2 Asian*  *5 Mixed* |
| *Light et. al.,*  *2017* | 7 | 11 | 4 | 10 | *not stated* |
| *O’Donnel et. al., 2023* | 5 | 26 | *no healthy control group* | | *26 AA*  *1 Hispanic*  *4 Other* |

**Supplementary table 1***. Reported Demographic Data per each study.*

**Section 3:** Quality assessment tool

Study quality was assessed using the Joanna Briggs Institute (JBI) Critical Appraisal Checklist for Randomized Controlled Trials (RCTs) (1) and for Quasi Experimental Studies. The JBI checklist was chosen due to its flexibility, broad applicability, and practical ease of use, particularly suitable when systematically reviewing diverse study designs, including both randomized and non-randomized medication trials (2). Given the absence of standardized critical appraisal tools addressing neuroimaging methodologies in psychiatric research, five supplementary neuroimaging-specific criteria were developed and agreed upon by author consensus. For transparency and clarity, individual appraisal items were qualitatively reported in the supplementary tables and text under section 3. Additionally, an overall quality score was determined by categorizing studies as 'high quality' (≥70% criteria met), 'moderate quality' (50–69%), or 'low quality' (<50%), facilitating consistent interpretation across studies, a method employed by previous systematic reviews (3-6). The score was calculated based on the percentage of Yes from the overall.

***Part A:*** *General study design based on JBI appraisal tools*

| **Question** | **YES** | **NO** | **UNCLEAR** | **N/A** |
| --- | --- | --- | --- | --- |
| Based on JBI for RCTs for the RCT studies | | | | |
| Q1. Was true randomization used for assignment of participants to treatment groups? |  |  |  |  |
| Q2. Was allocation to treatment groups concealed? |  |  |  |  |
| Q3. Were treatment groups similar at the baseline? |  |  |  |  |
| Q4. Were participants blind to treatment assignment? |  |  |  |  |
| Q5. Were those delivering the treatment blind to treatment assignment? |  |  |  |  |
| Q6. Were treatment groups treated identically other than the intervention of interest? |  |  |  |  |
| Q7. Were outcome assessors blind to treatment assignment? |  |  |  |  |
| Q8. Were outcomes measured in the same way for treatment groups? |  |  |  |  |
| Q9. Were outcomes measured in a reliable way? |  |  |  |  |
| Q10. Was follow up complete and if not, were differences between groups in terms of their follow up adequately described and analysed? |  |  |  |  |
| Q11. Were participants analysed in the groups to which they were randomized? |  |  |  |  |
| Q12. Was appropriate statistical analysis used? Including multiple corrections applied (neuroimaging data). |  |  |  |  |
| Q13. Was the trial design appropriate and any deviations from the standard RCT design accounted for in the conduct and analysis of the trial? |  |  |  |  |
| Based on JBI for quasi experimental studies for this type of studies | | | | |
| Is it clear in the study what is the ‘cause’ and what is the ‘effect’ (i.e., there is no confusion about which variable comes first)? |  |  |  |  |
| Were the participants included in any comparisons similar? |  |  |  |  |
| Were the participants included in any comparisons receiving similar treatment/care, other than the exposure or intervention of interest? |  |  |  |  |
| Was there a control group? |  |  |  |  |
| Were there multiple measurements of the outcome both pre and post the intervention/exposure? |  |  |  |  |
| Was follow up complete and if not, were differences between groups in terms of their follow up adequately described and analysed? |  |  |  |  |
| Were the outcomes of participants included in any comparisons measured in the same way? |  |  |  |  |
| Were outcomes measured in a reliable way? |  |  |  |  |
| Was appropriate statistical analysis used? |  |  |  |  |

***Part B:*** *Neuroimaging Related*

| **Question** | **YES** | **NO** | **UNCLEAR** | **N/A** |
| --- | --- | --- | --- | --- |
| **Q1. Is the choice of neuroimaging modality and its parameters well-justified for the research question?** |  |  |  |  |
| **Q2. Are the data acquisition procedures described in sufficient detail for reproducibility and justified?** |  |  |  |  |
| **Q3. Were the preprocessing and quality control steps appropriate and sufficiently described?** |  |  |  |  |
| Q4. Is the chosen experimental task (or paradigm) well-suited and adequately described to measure the hypothesized cognitive or neural process in the study, and does it include appropriate controls or baseline conditions? |  |  |  |  |
| Q5. Is the chosen acquisition time (scan duration for fMRI, MRS, ASL, or recording duration for EEG) adequately justified and appropriate for the study’s goals, considering factors like signal-to-noise ratio, participant comfort, and expected effect sizes? |  |  |  |  |
| Q6. Is the electrode placement/vowel placement covering well the ROI and justified for the research question? |  |  |  |  |

**Supplementary table 2.** *Representation of the quality assessment tool employed.*

**Section 4:** Critical Appraisal of Study Quality

The methodological quality of each included study was independently assessed by IV, LB, CC and VM using the critical appraisal checklist tool described above. Each component was rated as “Yes”, “No”, “Unclear, or “Not Applicable”. If needed, discrepancies were discussed between reviewers or by consulting a third author (RM) until consensus was reached. See **Supplementary Table 2, 3 and 4** for the detailed item-level assessments, corresponding non-bias percentage scores, and overall impressions. Among the 26 studies appraised, 22 employed an RCT design, whilst 4 can be described as quasi experimental designs, each study design being appraised with its corresponding JBI tool.

15 studies demonstrated a high level of methodological rigor and were considered to have a low risk of bias. 14 of these were RCTs and 1 quasy-experimental. The remaining 11 studies were appraised of moderate quality. Overall the studies do not seem to have major faults, but rather incomplete descriptions of their methodology and analysis, which is penalized by the tools. For example, a few studies provided limited details on how randomization and allocation concealment were conducted or whether assessors remained blinded throughout data analysis. The authors did state that randomization and blinding took place, but did not describe the method. As per the JBI tools, we marked them with U (unclear). Another example is the absence of multiple corrections for neuroimaging data, which some authors acknowledged as a limitation (7) or despite appropriate use of statistical tests, they lack clarity on testing assumptions, power analysis or reporting effect sizes such as Ota et. al., 2015, Ye et. al., 2019 and Zhuo et. al., 2020 (8-10).

Regarding the neuroimaging appraisal, out of the 26 studies appraised, 22 were rated as high quality based on the non-bias percentage score. The remaining 4 were rated as moderate quality due to incomplete or unclear descriptions of data acquisition and preprocessing procedures, and a lack of justification for their approach to defining regions of interest, particularly in EEG studies.

| **Study** | **Q1** | **Q2** | **Q3** | **Q4** | **Q5** | **Q6** | **Q7** | **Q8** | **Q9** | **Q10** | **Q11** | **Q12** | **Q13** | **Non-Bias Percentage** | **Impression on Quality** |
| --- | --- | --- | --- | --- | --- | --- | --- | --- | --- | --- | --- | --- | --- | --- | --- |
| Sehatpour et. al., 2023 (7) | U | U | N | Y | Y | Y | Y | Y | Y | Y | U | N | Y | 8/13 (62%) | Moderate |
| Govani et.al., 2023 (11) | Y | U | Y | Y | Y | Y | Y | Y | Y | Y | U | Y | Y | 11/13 (85%) | High |
| Wolf et.al., 2022 (12) | U | U | U | Y | Y | Y | U | Y | Y | Y | U | Y | Y | 8/13 (62%) | Moderate |
| Yang et.al., 2022 (13) | U | U | Y | U | U | Y | U | Y | Y | Y | U | Y | Y | 7/13 (54%) | Moderate |
| Schulteisis et. al., 2022 (14) | Y | U | N | Y | Y | Y | U | Y | Y | N | U | N | Y | 7/13 (54%) | Moderate |
| Guimares et.al., 2021 (15) | Y | U | Y | Y | Y | Y | U | Y | Y | N | U | U | Y | 8/13 (62%) | Moderate |
| Kantrowitz et.al., 2018 (16) | U | U | Y | Y | N | Y | Y | Y | Y | N | N | Y | Y | 8/13 (62%) | Moderate |
| McQueen et.al., 2018 (17) | U | U | U | Y | Y | Y | Y | Y | Y | N | N | Y | Y | 8/13 (62%) | Moderate |
| Chrysa et.al., 2018 (18) | U | U | Y | Y | Y | Y | Y | Y | Y | U | U | Y | Y | 9/13 (70%) | High |
| Strzelecki et. al., 2015 (19) | Y | Y | Y | Y | Y | Y | Y | Y | Y | U | U | U | Y | 10/13 (77%) | High |
| Strzelecki et. al., 2015 (20) | U | U | Y | Y | Y | Y | Y | Y | Y | U | U | Y | Y | 9/13 (70%) | High |
| Strzelecki et. al., 2015 (21) | U | U | Y | Y | Y | Y | Y | Y | Y | U | U | U | Y | 8/13 (62%) | Moderate |
| D’Souza et.al., 2018 (22) | Y | U | Y | Y | Y | Y | U | Y | Y | U | Y | Y | Y | 10/13 (77%) | High |
| Yurgelun et. al., 2005 (23) | Y | U | Y | Y | N | Y | N | Y | Y | U | Y | Y | Y | 9/13 (70%) | High |
| Forsyth et. al., 2017 (24) | Y | U | Y | Y | Y | Y | U | Y | Y | U | Y | Y | Y | 10/13 (77%) | High |
| McQueen et. al., 2020 (25) | Y | U | Y | Y | Y | Y | U | Y | Y | U | Y | Y | Y | 10/13 (77%) | High |
| Swerdlow et. al., 2024 (26) | Y | U | N | Y | Y | Y | Y | Y | Y | Y | Y | Y | Y | 11/13 (85%) | High |
| Molina et. al., 2020 (27) | U | U | N | Y | Y | Y | Y | Y | Y | U | Y | Y | Y | 9/13 (70%) | High |
| Greenwood et.al., 2018 (28) | Y | U | Y | Y | Y | N | Y | Y | Y | Y | Y | Y | Y | 11/13 (85%) | High |
| Giris et. al., 2019 (29) | Y | U | Y | Y | Y | Y | Y | Y | Y | U | Y | Y | Y | 11/13 (85%) | High |
| Light et. al, 2017 (30) | N | U | N | Y | Y | Y | Y | Y | Y | Y | Y | Y | Y | 10/13 (77%) | High |
| O’Donnell et. al., 2023 (31) | Y | U | U | Y | Y | Y | U | Y | Y | Y | Y | U | Y | 9/13 (70%) | High |

**Supplementary table 3.** *Quality Assessment Results Part A; Y=Yes, N=No, U=Unclear; These studies employed an RCT design and were appraised via the JBI tool for RCT studies.*

| **Study** | **Q1** | **Q2** | **Q3** | **Q4** | **Q5** | **Q6** | **Q7** | **Q8** | **Q9** | **Non-Bias Percentage** | **Impression on Quality** |
| --- | --- | --- | --- | --- | --- | --- | --- | --- | --- | --- | --- |
| Ota et. al., 2015 (8) | Y | Y | Y | N | N | Y | Y | U | U | 5/9 (56%) | Moderate |
| Ye et. al., 2019 (9) | Y | Y | Y | N | Y | Y | Y | U | U | 6/9 (67%) | Moderate |
| Zhuo et. al., 2020 (10) | Y | Y | Y | N | Y | U | Y | U | U | 5/9 (56%) | Moderate |
| Pillinger et. al. 2019 (32) | Y | Y | Y | Y | Y | Y | Y | Y | Y | 9/9 (100%) | High |

**Supplementary table 4***. Quality Assessment Results Part A; Y=Yes, N=No, U=Unclear, N/A=non-applicable. These studies employed an open-label single group pre post intervention design or within group parallel designs and were appraised via the JBI tool for quasi-experimental studies.*

**Part B:** Neuroimaging Related

| **Study** | **Q1** | **Q2** | **Q3** | **Q4** | **Q5** | **Q6** | **Overall Score** | **Impression on Quality** |
| --- | --- | --- | --- | --- | --- | --- | --- | --- |
| Sehatpour et. al., 2023 (7) | Y | Y | N | Y | Y | U | 4/6 (66%) | Moderate |
| Govani et.al., 2023 (11) | Y | Y | Y | Y | Y | Y | 6/6 (100%) | High |
| Wolf et.al., 2022 (12) | Y | Y | Y | Y | Y | Y | 6/6 (100%) | High |
| Yang et.al., 2022 (13) | Y | U | U | Y | Y | Y | 4/6 (66%) | Moderate |
| Schulteisis et. al., 2022 (14) | Y | Y | Y | Y | Y | Y | 6/6 (100%) | High |
| Guimares et.al., 2021 (15) | Y | Y | U | Y | Y | Y | 5/6 (83%) | High |
| Kantrowitz et.al., 2018 (16) | Y | Y | Y | Y | Y | Y | 6/6 (100%) | High |
| McQueen et.al., 2018 (17) | Y | Y | Y | Y | Y | Y | 6/6 (100%) | High |
| Chrysa et.al., 2018 (18) | Y | Y | Y | Y | Y | Y | 6/6 (100%) | High |
| Strzelecki et. al., 2015 (19) | Y | Y | Y | Y | Y | U | 5/6 (83%) | High |
| Strzelecki et. al., 2015 (20) | Y | Y | Y | Y | Y | Y | 6/6 (100%) | High |
| Strzelecki et. al., 2015 (21) | Y | Y | Y | Y | Y | Y | 6/6 (100%) | High |
| D’Souza et.al., 2018 (22) | Y | Y | Y | Y | Y | Y | 6/6 (100%) | High |
| Yurgelun et. al., 2005 (23) | Y | Y | Y | Y | Y | Y | 6/6 (100%) | High |
| Forsyth et. al., 2017 (24) | Y | Y | Y | Y | Y | Y | 6/6 (100%) | High |
| Ota et. al., 2015 (8) | Y | Y | N | N | Y | Y | 4/6 (66%) | Moderate |
| McQueen et. al., 2020 (25) | Y | Y | Y | Y | Y | Y | 6/6 (100%) | High |
| Ye et. al., 2019 (9) | Y | Y | Y | Y | U | Y | 5/6 (83%) | High |
| Zhuo et. al., 2020 (10) | Y | Y | Y | Y | Y | Y | 6/6 (100%) | High |
| Swerdlow et. al., 2024 (26) | Y | Y | Y | Y | Y | Y | 6/6 (100%) | High |
| Molina et. al., 2020 (27) | Y | Y | Y | Y | Y | Y | 6/6 (100%) | High |
| Greenwood et.al., 2018 (28) | Y | Y | Y | Y | Y | Y | 6/6 (100%) | High |
| Pillinger et. al. 2019 (32) | Y | Y | Y | Y | Y | Y | 6/6 (100%) | High |
| Giris et. al., 2019 (29) | Y | Y | Y | Y | Y | Y | 6/6 (100%) | High |
| Light et. al, 2017 (30) | Y | Y | Y | Y | Y | Y | 6/6 (100%) | High |
| O’Donnell et. al., 2023 (31) | Y | Y | U | Y | U | Y | 4/6 (66%) | Moderate |

**Supplementary table 5.** *Quality Assessment Results Part B; Y=Yes, N=No, U=Unclear;*

**References**

1. Barker TH, Stone JC, Sears K, Klugar M, Tufanaru C, Leonardi-Bee J, et al. The revised JBI critical appraisal tool for the assessment of risk of bias for randomized controlled trials. JBI Evidence Synthesis. 2023.

2. Aromataris E, Lockwood C, Porritt K, Pilla B, Jordan Z. JBI Manual for Evidence Synthesis2024.

3. Ferguson AA, Khan AI, Abuzainah B, Chaudhuri D, Khan KI, Al Shouli R, et al. Clinical Effectiveness of N-Methyl-D-Aspartate (NMDA) Receptor Antagonists in Adult Obsessive-Compulsive Disorder (OCD) Treatment: A Systematic Review. Cureus. 2023.

4. Schalinski I, Karimov-Zwienenberg M, Symphor W, Peraud W, Décamps G. Childhood trauma, PTSD/CPTSD and chronic pain: A systematic review. Plos One. 2024;19(8).

5. Dijkshoorn ABC, van Stralen HE, Sloots M, Schagen SB, Visser‐Meily JMA, Schepers VPM. Prevalence of cognitive impairment and change in patients with breast cancer: A systematic review of longitudinal studies. Psycho-Oncology. 2021;30(5):635-48.

6. Whittaker AL, George RP, O’Malley L. Prevalence of cognitive impairment following chemotherapy treatment for breast cancer: a systematic review and meta-analysis. Scientific Reports. 2022;12(1).

7. Sehatpour P, Iosifescu DV, De Baun HM, Shope C, Mayer MR, Gangwisch J, et al. Dose-Dependent Augmentation of Neuroplasticity-Based Auditory Learning in Schizophrenia: A Double-Blind, Placebo-Controlled, Randomized, Target Engagement Clinical Trial of the NMDA Glutamate Receptor Agonist d-serine. Biol Psychiatry. 2023;94(2):164-73.

8. Ota M, Wakabayashi C, Sato N, Hori H, Hattori K, Teraishi T, et al. Effect of L-theanine on glutamatergic function in patients with schizophrenia. Acta Neuropsychiatr. 2015;27(5):291-6.

9. Ye J, Lin X, Jiang D, Chen M, Zhang Y, Tian H, et al. Adjunct ketamine treatment effects on treatment-resistant depressive symptoms in chronic treatment-resistant schizophrenia patients are short-term and disassociated from regional homogeneity changes in key brain regions – a pilot study. Psychiatry and Clinical Psychopharmacology. 2019;29(4):907-15.

10. Zhuo C, Lin X, Tian H, Liu S, Bian H, Chen C. Adjunct ketamine treatment of depression in treatment-resistant schizophrenia patients is unsatisfactory in pilot and secondary follow-up studies. Brain Behav. 2020;10(5):e01600.

11. Govani V, Shastry A, Iosifescu D, Govil P, Mayer M, Sobeih T, et al. Augmentation of learning in schizophrenia by D-serine is related to auditory and frontally-generated biomarkers: A randomized, double-blind, placebo-controlled study. Res Sq. 2023.

12. Wolf DH, Zheng D, Kohler C, Turetsky BI, Ruparel K, Satterthwaite TD, et al. Effect of mGluR2 positive allosteric modulation on frontostriatal working memory activation in schizophrenia. Mol Psychiatry. 2022;27(2):1226-32.

13. Yang YS, Maddock RJ, Zhang H, Lee J, Hellemann G, Marder SR, et al. N-Acetylcysteine effects on glutathione and glutamate in schizophrenia: A preliminary MRS study. Psychiatry Res Neuroimaging. 2022;325:111515.

14. Schultheis C, Rosenbrock H, Mack SR, Vinisko R, Schuelert N, Plano A, et al. Quantitative electroencephalography parameters as neurophysiological biomarkers of schizophrenia-related deficits: A Phase II substudy of patients treated with iclepertin (BI 425809). Transl Psychiatry. 2022;12(1):329.

15. Guimaraes TM, Guimaraes MRC, Oliveira IAF, Leoni RF, Santos AC, Dursun SM, et al. Mononitrate Isosorbide as an Adjunctive Therapy in Schizophrenia: A Randomized Controlled Crossover Trial. J Clin Psychopharmacol. 2021;41(3):260-6.

16. Kantrowitz JT, Epstein ML, Lee M, Lehrfeld N, Nolan KA, Shope C, et al. Improvement in mismatch negativity generation during d-serine treatment in schizophrenia: Correlation with symptoms. Schizophr Res. 2018;191:70-9.

17. McQueen G, Lally J, Collier T, Zelaya F, Lythgoe DJ, Barker GJ, et al. Effects of N-acetylcysteine on brain glutamate levels and resting perfusion in schizophrenia. Psychopharmacology (Berl). 2018;235(10):3045-54.

18. Retsa C, Knebel JF, Geiser E, Ferrari C, Jenni R, Fournier M, et al. Treatment in early psychosis with N-acetyl-cysteine for 6months improves low-level auditory processing: Pilot study. Schizophr Res. 2018;191:80-6.

19. Strzelecki D, Podgorski M, Kaluzynska O, Gawlik-Kotelnicka O, Stefanczyk L, Kotlicka-Antczak M, et al. Supplementation of Antipsychotic Treatment with the Amino Acid Sarcosine Influences Proton Magnetic Resonance Spectroscopy Parameters in Left Frontal White Matter in Patients with Schizophrenia. Nutrients. 2015;7(10):8767-82.

20. Strzelecki D, Podgorski M, Kaluzynska O, Stefanczyk L, Kotlicka-Antczak M, Gmitrowicz A, et al. Adding Sarcosine to Antipsychotic Treatment in Patients with Stable Schizophrenia Changes the Concentrations of Neuronal and Glial Metabolites in the Left Dorsolateral Prefrontal Cortex. Int J Mol Sci. 2015;16(10):24475-89.

21. Strzelecki D, Podgórski M, Kałużyńska O, Gawlik-Kotelnicka O, Stefańczyk L, Kotlicka-Antczak M, et al. Supplementation of antipsychotic treatment with sarcosine – GlyT1 inhibitor – causes changes of glutamatergic 1NMR spectroscopy parameters in the left hippocampus in patients with stable schizophrenia. Neuroscience Letters. 2015;606:7-12.

22. D'Souza DC, Carson RE, Driesen N, Johannesen J, Ranganathan M, Krystal JH, et al. Dose-Related Target Occupancy and Effects on Circuitry, Behavior, and Neuroplasticity of the Glycine Transporter-1 Inhibitor PF-03463275 in Healthy and Schizophrenia Subjects. Biol Psychiatry. 2018;84(6):413-21.

23. Yurgelun-Todd DA, Coyle JT, Gruber SA, Renshaw PF, Silveri MM, Amico E, et al. Functional magnetic resonance imaging studies of schizophrenic patients during word production: effects of D-cycloserine. Psychiatry Res. 2005;138(1):23-31.

24. Forsyth JK, Bachman P, Mathalon DH, Roach BJ, Ye E, Asarnow RF. Effects of Augmenting N-Methyl-D-Aspartate Receptor Signaling on Working Memory and Experience-Dependent Plasticity in Schizophrenia: An Exploratory Study Using Acute d-cycloserine. Schizophr Bull. 2017;43(5):1123-33.

25. McQueen G, Lay A, Lally J, Gabay AS, Collier T, Lythgoe DJ, et al. Effect of single dose N-acetylcysteine administration on resting state functional connectivity in schizophrenia. Psychopharmacology (Berl). 2020;237(2):443-51.

26. Swerdlow NR, Gonzalez CE, Raza MU, Gautam D, Miyakoshi M, Clayson PE, et al. Effects of Memantine on the Auditory Steady-State and Harmonic Responses to 40 Hz Stimulation Across Species. Biol Psychiatry Cogn Neurosci Neuroimaging. 2024;9(3):346-55.

27. Molina JL, Voytek B, Thomas ML, Joshi YB, Bhakta SG, Talledo JA, et al. Memantine Effects on Electroencephalographic Measures of Putative Excitatory/Inhibitory Balance in Schizophrenia. Biol Psychiatry Cogn Neurosci Neuroimaging. 2020;5(6):562-8.

28. Greenwood LM, Leung S, Michie PT, Green A, Nathan PJ, Fitzgerald P, et al. The effects of glycine on auditory mismatch negativity in schizophrenia. Schizophr Res. 2018;191:61-9.

29. Girgis RR, Baker S, Mao X, Gil R, Javitt DC, Kantrowitz JT, et al. Effects of acute N-acetylcysteine challenge on cortical glutathione and glutamate in schizophrenia: A pilot in vivo proton magnetic resonance spectroscopy study. Psychiatry Res. 2019;275:78-85.

30. Light GA, Zhang W, Joshi YB, Bhakta S, Talledo JA, Swerdlow NR. Single-Dose Memantine Improves Cortical Oscillatory Response Dynamics in Patients with Schizophrenia. Neuropsychopharmacology. 2017;42(13):2633-9.

31. O’Donnell P, Dong C, Murthy V, Asgharnejad M, Du X, Summerfelt A, et al. The D-amino acid oxidase inhibitor luvadaxistat improves mismatch negativity in patients with schizophrenia in a randomized trial. Neuropsychopharmacology. 2023;48(7):1052-9.

32. Pillinger T, Rogdaki M, McCutcheon RA, Hathway P, Egerton A, Howes OD. Altered glutamatergic response and functional connectivity in treatment resistant schizophrenia: the effect of riluzole and therapeutic implications. Psychopharmacology (Berl). 2019;236(7):1985-97.
